# Towards Optimization of Machining Performance and Sustainability Aspects when Turning AISI 1045 Steel under Different Cooling and Lubrication Strategies

**DOI:** 10.3390/ma12183023

**Published:** 2019-09-18

**Authors:** Adel T. Abbas, Faycal Benyahia, Magdy M. El Rayes, Catalin Pruncu, Mohamed A. Taha, Hussien Hegab

**Affiliations:** 1 Department of Mechanical Engineering, College of Engineering, King Saud University, Riyadh 11421, Saudi Arabia; 2 Mechanical Engineering, Imperial College London, London SW7 2AZ, UK; 3 Mechanical Engineering Department, University of Birmingham, Birmingham B15 2TT, UK; 4 Department of Mechanical Design and Production, Zagazig University, Zagazig 44519, Egypt; 5 Mechanical Design and Production Engineering Department, Cairo University, Giza 12613, Egypt

**Keywords:** AISI 1045 steel, machining, optimization, cooling, lubrication, sustainability

## Abstract

In this work, an extensive analysis has been presented and discussed to study the effectiveness of using different cooling and lubrication techniques when turning AISI 1045 steel. Three different approaches have been employed, namely dry, flood, and minimum quantity lubrication based nanofluid (MQL-nanofluid). In addition, three multi-objective optimization models have been employed to select the optimal cutting conditions. These cases include machining performance, sustainability effectiveness, and an integrated model which covers both machining outputs (i.e., surface roughness and power consumption) and sustainability aspects (carbon dioxide emissions and total machining cost). The results provided in this work offer a clear guideline to select the optimal cutting conditions based on different scenarios. It should be stated that MQL-nanofluid offered promising results through the three studied cases compared to dry and flood approaches. When considering both sustainability aspects and machining outputs, it is found that the optimal cutting conditions are cutting speed of 147 m/min, depth of cut of 0.28 mm and feed rate of 0.06 mm/rev using MQL-nanofluid. The three studied multi-objective optimization models obtained in this work provide flexibility to the decision maker(s) to select the appropriate cooling/lubrication strategy based on the desired objectives and targets, whether these targets are focused on machining performance, sustainability effectiveness, or both. Thus, this work offers a promising attempt in the open literature to optimize the machining process from the performance–sustainability point of view.

## 1. Introduction

The AISI 1045 steel is a common material used in manufacturing sector, which is employed as a tool or workpiece. The main machine components manufactured from 1045 are shafts, gears, crankshafts, connecting rods, bolts, etc. [1]. Even though the manufacturing of this material seems quite established, the need for high productivity combined with superior sustainability pushes the research community to identify novel machining techniques. These are based on friendly lubrication as well as innovative optimization techniques. In the open literature [2], the machining of AISI 1045 steel was simulated using Abaqus-FEM package to detect the optimum parameters (i.e., cutting forces, feed rate and cutting depth) against coated cutting tools made of carbide coatings. To better predict machining performance, Devotta et al. [3] investigated the effect of rake angle on chip segmentation. In the experimental routine, the behavior of tool–chip interface was studied by [4] considering uncoated tungsten inserts. To understand the formation of serrated chips, Zhang et al. [5] machined a hardened AISI 1045 steel without any lubrication. They linked the chips’ breakability to the adiabatic shear process. Furthermore, Zhang and Wu [6] showed that a higher speed of machining can produce a superior chip serration. It was also shown that a higher value of cutting speed can promote a slightly lower value of the cutting force, which can imply lower energy consumption [7]. In addition, a higher cutting speed promotes lower surface finish which in turn may lead to a local increase of tool–chip contact temperature [8]. By applying a microgroove texture on the cutting tools, an optimized tool life can be achieved. The longer tool life allows a reduced energy consumption during machining of AISI 1045 steel because there is no need for replacing the cutting tip. The performance of a designed groove depends mainly on the width, groove depth, and edge distance [9]. Some micro-capillary networks manufactured in the cutting tool surfaces may significantly affect the machining forces and improve the friction conditions in dry machining [10]. Recently, Kong and Wang [11] explored the performance of elliptical vibration cutting (EVC) against a typical machining process while employing AISI 1045 steel. They found that the cutting force, residual stresses, and local temperature were lower in EVC compared to conventional cutting, which then brings only a limited amount of residual stress.

Davies et al. [12] highlighted the need of a more predictive strategy, rather than interpretive simulation, in order to improve the tool design used in cutting. It should be stated that a better prediction can help to control the contact cutting tools which further promote lower cutting force and/or thrust force that can finally reduce the energy necessary for machining [13]. Wu et al. [14] proposed a modified damage mechanistic model to improve the turning of AISI 1045 steel that enables a better chip breakage. The classical Taguchi strategy design was applied to detect the optimum combination between input parameters (i.e., cutting speed, cutting depth, and feed rate) to improve the output results (i.e., surface roughness) [1]. Ahmad et al. [15] studied surface roughness (SR) during machining of AISI 1045 steel by applying different approximation/optimization techniques (i.e., response surface methodology (RSM), multi-objective optimization, fuzzy logic modelling). They found a highly good agreement between experiments and optimization process (99.3% similarity in the results). The best SR was obtained using particle swarm optimization while faster results were achieved applying genetic algorithm. An optimization machining process was introduced by combining minimum quantity lubrication (MQL) with considering the nanofluid as lubricant and grey relational analysis (GRA) as an optimization technique. It was found that only a limited amount of improvement was achieved (around 4.32%) [16]. Furthermore, in the open literature [17,18] some analytical constitutive relations were proposed to predict the machining temperatures generated when turning AISI 1045 steel. The computed model was very efficient, which permits real-time prediction and reduced experimental complexity for the input variables.

Minimum quantity cooling lubrication (MQCL) has been applied during turning of AISI 1045, which helps to reduce the amount of plastic deformations. This process further allows to decrease the surface crumple zone by a large amount (~50%) when compared with dry cutting [19]. A significant element in this process is the nozzle position, which directly affects the output parameters [20]. A substantial contribution can be achieved, with longer tool wear and superior cutting performance, when a suitable nozzle distance is determined during MQL [21]. The hybrid methods that combine MQL and ultrasonic vibration (UV) were engaged successfully to improve cutting condition and extend tool life [22]. Different graphite oil-based nanofluids were applied during MQL in order to reduce the cutting force and temperature [23], which in turn offer better machining performance when compared with typical MQL. Overall, it was noted that minimum quantity lubrication (MQL) can lead to increased cost-effectiveness for the overall machining process [24]. Govindaraju et al. [25] explored the performance of liquid nitrogen (LN_2_) as coolant compared against a conventional coolant. They showed that the cutting temperature (CT) may be reduced by up to 51%, while at the same time it is possible to increase the feed rate. However, this can generate a higher thrust force due to a very high local temperature at the tool interface [26]. Different studies [27,28,29,30] proved that using MQL-nanofluid was effective in improving overall machinability, as it supports the tribology and heat transfer of the cutting operations.

It is clearly obtained in the open literature [31] that elimination of the negative effect of coolants or lubricants can be achieved by replacing them with alternative biodegradable coolants such as vegetable oils. In addition, it is found that using advanced sustainable techniques in cutting operations (i.e., dry cutting without coolants, methods using the minimal amounts of coolants and lubricants, cryogenic cooling, or cooling with high-pressure feeding of lubricating fluid) offers a promising guideline for establishing a sustainable machining environment. Moreover, to achieve a sustainable manufacturing process it is very important to understand and define the concepts and needs related to the sustainability approach [32]. Thus, this work does not only focus on the machining performance, but also considers the sustainability aspects.

Despite a large amount of research, there is not a systematic study regarding the optimization techniques and lubrication conditions that offers a reliable machining condition for AISI 1045 steel with superior performances. This study presents a carefully evaluation of turning AISI 1045 steel by applying robust optimization algorithm based on genetic programming approach correlated to modern different cooling and lubrication strategies, namely dry, flood, and MQL-nanofluids. In addition, three different models have been presented and discussed in this work (i.e., machining performance, sustainability effectiveness, and the integrated model includes both). The results gathered in this research are very promising and can be used successfully for industrial application.

## 2. Experimentation and Methodology

**Figure 1:**
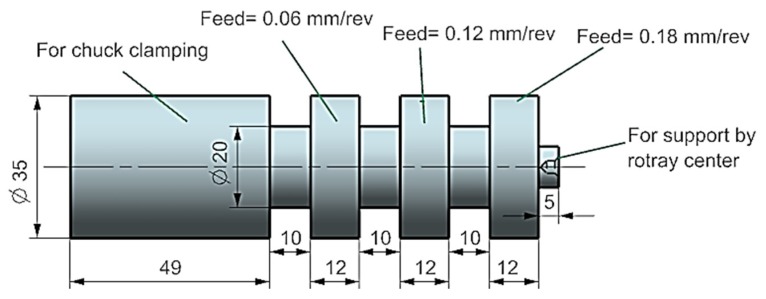
AISI 1045 specimen used for experimentation.

This study uses AISI 1045 steel specimens of 130 mm and 35 mm initial diameters. The specimens’ geometry is shown in Figure 1 while their material chemical composition is detailed in Table 1. The specimens were first heated to 840 °C until uniform temperature was reached, then were soaked for an hour. After soaking they were quenched in water, generating a martensitic structure. This phase was followed by a hardening then a tempering stage through heating the specimens up to 600 °C until a uniform temperature distribution was obtained then soaking them for two hours and finally cooling them in still air. Once this heat treatment was done, the average hardness and tensile strength of specimens were of HV243 and 635 MPa, respectively. In terms of the machine tool and cutting inserts, the cutting operations of specimens were conducted using a CNC turning center with Sinumeric 840-D control system supported by a motor power of 13 kW (Emco, Hallien, Austria). The tool holder (SDJCL 2020 K11, Sandvik, Sweden)) clamped a cutting uncoated tungsten carbide insert (Sandvik, Sweden) of type VBMT 160404-UM characterized by a clearance angle of 7°, a nose radius of 0.4 mm, a cutting-edge angle of 55° and a rake angle of 6°. Based on a Taguchi orthogonal array design of experiment, a total number of 81 (3^4^) tests were performed to investigate the effect of four parameters (i.e., 27 tests for each cooling strategy), each varied over three levels, on the machining quality. The studied parameters and their related tested levels are provided in Table 2. It should be stated that the cutting conditions were selected based on the recommended information of the used cutting inserts as well as the available information in the open literature. L27 orthogonal array was used for cutting experiments of each strategy as provided in Table 3. 

**Figure 2:**
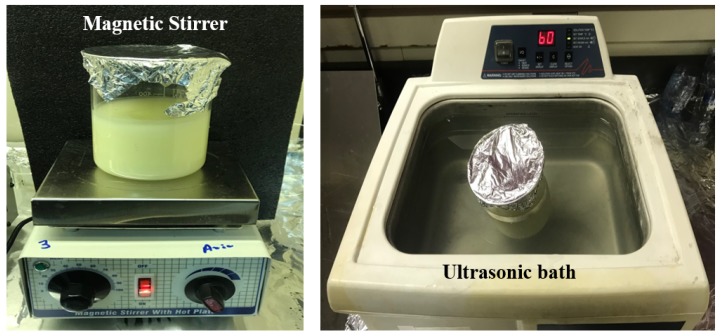
Magnetic stirrer and ultrasonic bath used to disperse Al_2_O_3_ nano-additives in the vegetable oil.

The lubricating fluid used in the conventional cooling was made of a volume of lubricant oil (type ECO-COOL-MK-3, made by Saudi petroleum company, KSA) diluted and homogenized in five equivalent volumes of distillated water. This lubricant was directed to the workpiece through two nozzles, one of which was directed to the tool tip, and the other was directed to the workpiece surface. The fluid was operated by a 2.2 kW pump, connected to a 230-liter tank, at a flow rate of 160 L/h. Regarding the MQL-nanofluid, the nanofluid was made of vegetable oil mixed with nanoparticles. Aluminum oxide (Al_2_O_3_) nanoparticles with a size of 30 nm were used. The physicochemical properties of the used vegetable base oils were as follows: kinematic viscosity of 401C (cSt: 40.05), viscosity index of 206, flashpoint (0 °C) of 252, and pour point (0 °C) of –12.00. To avoid clogging while preparing the nanofluid, as recommended by literature, a concentration of 1 wt.% of nanoparticles in the vegetable base oils was used. The mixture was then ultra-sonicated at high frequency (40 kHz sonicator—Cole-Parmer 8893, COLE PARMER, USA) for about 60 min followed by magnetic stirring (Figure 2) for 30 min. These preparation steps were repeated until the nanoparticles were uniformly distributed in the vegetable oil. The obtained nanofluid was stable when no settlement of particles took place during the machining process.

**Figure 3:**
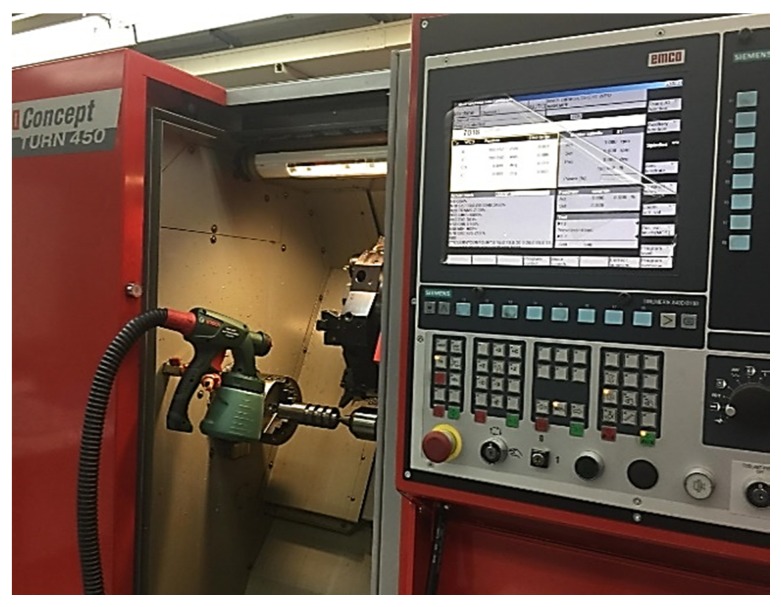
The experimental setup view.

The nanofluid was operated using a Bosch spray pump PFS1000, Bosch, Germany) having a power input of 410 W and adjustable flow rate 0–100 mL/min. The flow rate adopted was 120 mL/h. Figure 3 shows the test rigs for conventional and MQL-nanofluid machining conditions. In conformity to literature recommendations [33], during the experimental tests, certain safety procedures (i.e., standard nano-additives safety datasheet) were reinforced to maintain standard health and safety levels in the workshop and for the operator. A standard ventilator was placed in the workspace to get rid of the resultant nano-mist in the surroundings. Also, the disposal of the nanofluid was practiced according to the standard material safety datasheets. In fact, the nanofluid was carefully filtered before being released to the sewer.

The results of this study are evaluated based on two main machining responses: the surface roughness and the power consumption. The surface roughness was measured using a Tesa-Rougsurf-90G made by TESA-Switzerland, while the power consumption was processed through Equation (1):

(1) $$\mathrm{Power}=\mathrm{Voltage}*\mathrm{Current}*\sqrt{3}\;\mathrm{Cos}\;ø.$$

Two power meters (type: Tactix 403057, Tactix, China) were connected to the power supply of the machine to measure the voltage and the current powering the machine. Thus, the measured power is only for the machine tool. According to its datasheet, the machine was a balanced three phase load, and hence the current was measured over one phase (Ammeter) and the voltage was measured between the two other phases (Voltmeter). The load power factor was provided by the datasheet and the equipment accuracy was 0.02 Ampere. During each cut, three separate readings were taken and recorded in an excel sheet where the power could be calculated. 

In addition, this work considers two sustainable indicators, namely overall machining costs and CO_2_ emissions. In order to determine the overall cost, several elements were considered: machine tool cost, cutting tooling cost, materials cost, conventional fluid cost, MQL setup cost, and nanofluid preparation cost. Regarding the calculations of the CO_2_ emissions, this depended on the power consumed in each cutting test using a standard defined emission intensity [33].

**Figure 4:**
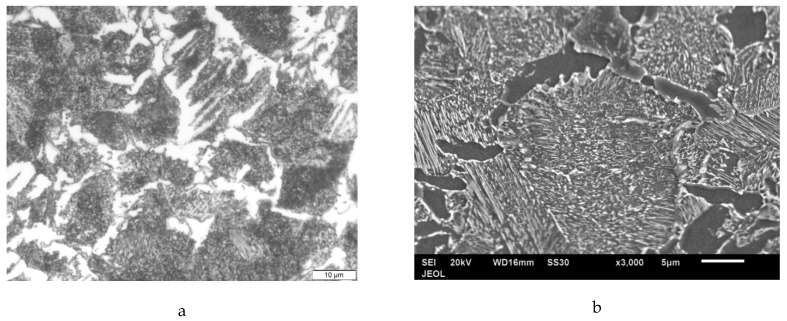
(**a**) Optical micrograph of tempered steel it is composed of ferrite (white) and tempered martensite (dark). (**b**) Secondary electron image of the dark area in optical micrograph (tempered martens).

It should also be stated that the hardening treatment conducted on the as-received material increased the hardness range from (185–190 HV) to (240–246 HV), which consequently could improve the surface quality of the machined material. Increasing hardness, i.e., reducing ductility, reduces the material’s plastic flow capacity, and thus offering a better surface finish. It should be stated that all hardness measurements were captured on the cylindrical surfaces with a diameter of 35 mm, and the mean value was considered. In addition, the difference in the hardness values along the machined surface was very small, and the hardness was almost uniform. A brittle interaction between a cutting tool and a low-ductility (hard) material induces separation during machining rather than a plastic flow which may cause surface irregularities. It is already known that the microstructure of annealed/normalized AISI 1045 is composed of ferrite and pearlite. The microstructure of tempered specimen is shown in Figure 4a, which is composed of ferrite areas (white) and tempered martensite (dark) as observed by optical microscope (Olympus, Japan). The same microstructure was enlarged using SEM as shown Figure 4b, in which the ferrite appears as the dark phase and carbides as white one.

## 3. Results and Discussions

**Figure 5:**
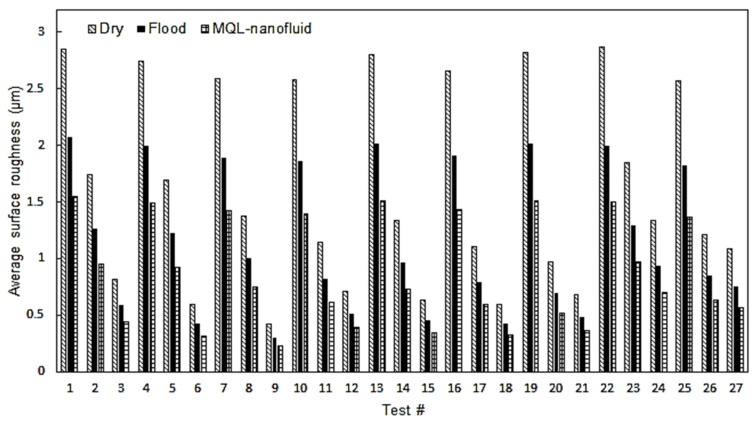
The surface roughness results.

In this section, the results associated with turning AISI 1045 under different cooling and lubrication techniques are provided. The surface roughness results are depicted in Figure 5. It can be observed that MQL-nanofluid approach offered the best performance in terms of the quality of the machined surface compared to the dry and flood techniques. In addition, the flood offered a better performance than dry machining trials. This is mainly attributed to the cooling capabilities of both flood and MQL-nanofluid techniques to reduce the severity of the high heat generated in the cutting zone. It should be also stated that MQL-nano-mist does not only offer a promising cooling property, but also it has a pure tribological effect on the performance of the cutting process. The nano-mist serves as rollers in the cutting zone, which affects and improves the friction behavior, and hence a better surface quality was observed when machining under MQL-nanofluid [32,33,34]. The lowest value of surface roughness was noticed in cutting test No. 9, which was performed at cutting speed of 150 m/min, feed rate of 0.06 mm/rev, and depth of cut of 0.25 mm under MQL-nanofluid. In addition, the lowest roughness values during both dry and flood techniques have been noticed at the same cutting test, as this test was performed at the lower levels of both feed rate and depth of cut. An improvement of 34.5% was observed when comparing the MQL-nanofluid with the flood techniques in cutting test No. 9. When comparing the MQL-nanofluid with the dry technique, an enhancement of 85.5% was noted. 

**Figure 6:**
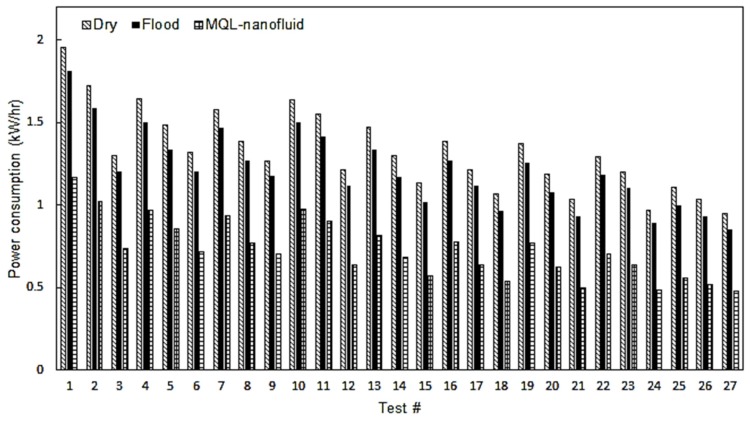
The power consumption results.

The power consumption results are provided in Figure 6. It can be also observed that MQL-nanofluid approach offered the best performance compared to the dry and flood techniques, and the flood offered a better performance than dry machining trials. This is due the significant effect of the employed mist which reduces the severe rubbing between the cutting tool and workpiece materials. In addition, the applied mist prevents the thermal shock mechanism and accordingly balances the thermal softening occurring to the cutting insert [35,36,37,38,39,40,41]. Thus, lower cutting forces and power consumption values are observed when using MQL-nanofluid, compared to the dry and flood techniques. The lowest value of power consumption was noticed in cutting test No. 27, which was performed at cutting speed of 100 m/min, feed rate of 0.06 mm/rev, and depth of cut of 0.25 mm under MQL-nanofluid. In addition, the lowest power consumption values during both dry and flood techniques have been noticed at the same cutting test, as this test was performed at the lower levels for all studied design variables. An improvement of 80.5% was observed when comparing the MQL-nanofluid with the flood techniques in cutting test No. 9, while comparing the MQL-nanofluid with the dry technique showed an enhancement of 99.5%. Since the power consumption results are directly proportional with the calculated CO_2_ emissions, it should be stated that MQL-nanofluid can be considered as an effective sustainable approach as it supports in decreasing the consumed power as well as the CO_2_ emissions (i.e., promising improved environmental impact).

**Figure 7:**
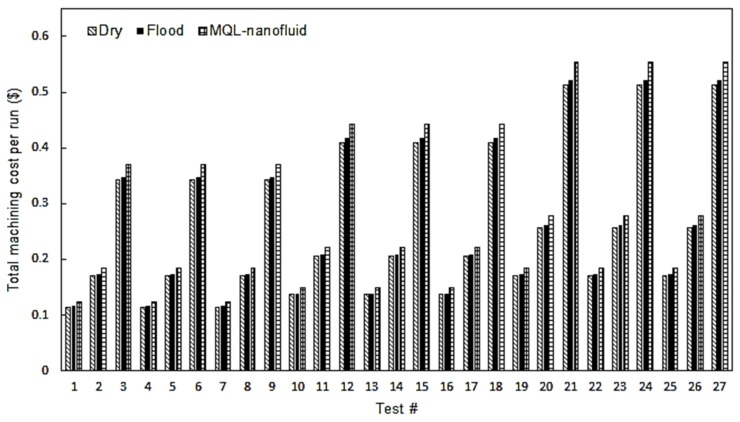
The total machining cost results.

In terms of the total machining costs, the results for the three approaches are provided as shown in Figure 7. It is observed that dry cutting approach offers the lowest total cost compared to the flood and MQL-nanofluid. That is mainly because of eliminating the costs related to the cutting fluid application. In addition, using flood coolant showed less machining costs compared to MQL-nanofluid technique. This is mainly due to the expensive costs related the preparation of the used nanofluid. Thus, it can be observed that using the dry approach can offer effective sustainable effects, especially when considering the machining costs.

The lowest machining cost was noticed in cutting test No. 7, which was performed at cutting speed of 150 m/min, feed rate of 0.18 mm/rev, and depth of cut of 0.25 mm under dry conditions. Similarly, the lowest machining costs for both MQL-nanofluid and flood techniques have been noticed in the same cutting test. A reduction of 7.9% was observed when comparing the dry cutting with MQL-nanofluid tests, while a slight reduction has been noticed when comparing the dry with flood coolant. 

In the next section three multi-objective optimization models are established in order to optimize the machining responses effects and the studied sustainable indicators.

## 4. Modeling and Multi-Objective Optimization

In order to establish the multi-objective optimization process, accurate models should be constructed to express the measured outputs and studied sustainable indicators in terms of the included design variables. A genetic programming approach was used to perform the modeling step. Genetic programming (GP) is one of the techniques based on the artificial intelligence. It is widely used in developing computer programs [40]. The selection process from nature is adopted through the GP to provide the best solutions. Every computer program is presented as the tree structure that consists of the nodes of the terminals and functions. The terminals are the inputs of the program and the functions may include arithmetic operations, standard programming functions and standard mathematical functions. In addition, every program (tree) is considered as a chromosome and the fitness of each chromosome is determined by the error between the program’s output and the actual output of the training set. GP manipulates the programs by applying the genetic algorithm operators such as crossover and mutation to produce new offspring. Several attempts have been made to apply the GP technique to model the machining processes [42,43,44]. In this study, the genetic programming technique was used to develop a symbolic regression between machining process responses and the design variables. The terminals consisted of the design variables and the functions included arithmetic and mathematical operations (+, -, ×, /, ^). In addition, the three test sets of using dry, flood and MQL-nanofluid were used as the raw data. The proposed models for using dry, flood and MQL-nanofluid are provided in Equations (2)–(5), (6)–(9) and (10)–(13), respectively. The goodness of fit and the correlation coefficient of all proposed models were greater than 0.999. Moreover, the highest mean square error among all proposed models was 2.5 × 10^-5^.

The non-dominated sorting genetic algorithm (NSGA-II) is employed in this study to perform the multi-objective optimization purpose. The NSGA-II [45,46] is one of the popularly used evolutionary multi-objective optimization algorithm, which attempt to find optimal Pareto front solutions. This technique is mainly based on using an elitist principle, and an explicit diversity preserving mechanism, called crowding distance. The evolutionary operators of the NSGA-II are based on genetic algorithms, which are namely crossover, mutation, and selection.

(2) $$\begin{array}{cc} {\mathrm{Ra}}_{\mathrm{dry}}=0.38+ & \left(4.38*d*f\right)+\left(763.95{*f}^{4}\right)+\left(34.23{*f}^{2}\right)+\left(13.43*d*f\right)-({f*d}^{2}\\ & *{\left(34.23*f\right)}^{d})), \end{array}$$

(3) $$\begin{array}{c} {\mathrm{Power}}_{\mathrm{dry}}=\left(0.27\right)+\left(0.139*d\right)+\left(0.005*v\right)+\left(0.022*v*f\right)+\left(0.0003{*f*v}^{2}{*d}^{2}\right)\\ -\left(0.00015{*d*f*v}^{2}\right), \end{array}$$

(4) $$\begin{array}{c} {\mathrm{CO}}_{2,\;\mathrm{dry}}=\left(0.12\right)+\left(0.002*v\right)+\left(0.0001{*f*v}^{2}\right)+\left(0.014{*v*f*d}^{2}\right)-\\ \left(d*2.35^{d}{*f}^{2}\right)-\left(4.36*10^{-7}{*f*v}^{3}\right), \end{array}$$

(5) $$\begin{array}{c} {\mathrm{Cos}t}_{\mathrm{dry}}=\left(0.011\right)+\left(2.01*10^{-5}*v\right)+\left(\frac{7.99}{v}\right)-\left(\frac{0.00011}{f^{2}}\right)-\left(\frac{0.0107}{{v*f}^{3}}\;\right)+\left(\frac{0.34}{{v*f}^{2}}\right)+\\ \left(1.007*10^{-9}*\frac{v}{f^{4}}\right)-\left(0.0003*v*f\right), \end{array}$$

(6) $$\begin{array}{cc} {\mathrm{Ra}}_{\mathrm{flood}}=\left(28.22\right) & +\left(0.603*v*f\right)+\left(0.015*v*d\right)+\left(559.83{*f}^{2}\right)-\left(0.034*v\right)\\ & -\left(1.84{*d}^{2}\right)-\left(22.75{*v}^{f}\right), \end{array}$$

(7) $$\begin{array}{c} {\mathrm{Power}}_{\mathrm{flood}}=\left(0.25\right)+\left(0.73*f\right)+\left(0.005*v\right)+\left(7.299*10^{-5}{*v}^{2}*\frac{f^{2}}{d}\right)+\\ \left(1.007*d*f*{\left(0.0002{*v}^{2}\right)}^{d}\right). \end{array}$$

(8) $$\begin{array}{cc} {\mathrm{CO}}_{2,\;\mathrm{flood}}=\left(0.14\right) & +\left(0.004*v\right)-\left(\frac{0.04}{d}\right)+\left(1.073*d*f\right)+\left(2.9*10^{-5}{*f*v}^{2}\right)\\ & +\left(3.21*10^{-7}{*v}^{3}{*d}^{2}\right)-\left(5.39*10^{-5}{*d*v}^{2}\right), \end{array}$$

(9) $$\begin{array}{c} {\mathrm{Cos}t}_{\mathrm{flood}}=\left(\frac{0.46}{v}\right)+\left(\frac{3.12}{v*f}\right)+{\left(2.068*10^{-5}*\left(\frac{28.35*v*f}{{f*v}^{2}-0.56^{{v*f}^{2}}}\right)-\left(v*0.56^{{v*f}^{2}}\right)\right)}^{-2.73}-\\ \left(0.004\right), \end{array}$$

(10) $$\begin{array}{c} {\mathrm{Ra}}_{\mathrm{MQL}}=\left(4.21\right)+\left(0.43*v*f\right)+\left(268.61{*f}^{2}\right)+\left(0.0001{*d*v}^{2}\right)-\left(0.03*v\right)-\\ \left(59.95*f\right)-\left(0.013*v*d\right)-\left(1.68{*v*f}^{2}\right), \end{array}$$

(11) $$\begin{array}{c} {\mathrm{Power}}_{\mathrm{MQL}}=\left(0.78\right)+\left(4.19*10^{-5}{*v}^{2}\right)+\left(2.86{*f*d}^{2}\right)+\left(8.23*10^{-5}{*f*v}^{2}\right)-\\ \left(0.007*v\right)-\left(14.4487*10^{10}{*d}^{v}\right)-\left(71.33*10^{10}{*f*d}^{v}\right), \end{array}$$

(12) $$\begin{array}{c} {\mathrm{CO}}_{2,\;\mathrm{MQL}}=\left(0.08\right)+\left(0.003*v*f\right)+\left(6.42*10^{-16}{*v}^{2}\right)+\left(0.008{*v*f*d}^{2}\right)-\\ \left(2.84*10^{-16}{*v}^{6}*\frac{d^{3}}{f}\right), \end{array}$$

(13) $$\begin{array}{c} {\mathrm{Cos}t}_{\mathrm{MQL}}=\left(0.008*f\right)-\left(2.86*10^{-15}{*v}^{4}\right)-\left(0.0014\right)+\\ \left(\frac{0.027}{6.42*10^{-14}+0.008*v*f+6.42*10^{-14}{*v}^{6}{*f}^{4}}\right). \end{array}$$

Regarding the NSGA-II, the population initialization is the first step. Secondly, non-dominated sorting is utilized to formulate the fronts. Consequently, non-dominated individuals of the population are assigned to be in the first front. The second front includes the dominated solutions by the first front, and so on. After defining all fronts, the individuals inside the lowest front rank are arranged by the crowding distance sorting to fit this front to be in the parent population. The individuals with higher crowding distance are promising to be in the parent population for keeping highly diverse individuals. The tournament selection, crossover, and mutation processes are carried out to produce new offspring population. All individuals in the parent population and offspring population compete to be inserted in the next generation. These steps are repeated until reaching the stopping criterion. The Pareto front is considered as the highest ranked front after meeting the stop criterion.

The GP machining characteristics models were used as the objective functions for the genetic algorithm. The values of the genetic algorithm (GA) parameters were: population size of 300, mutation factor of 0.012, and cross over rate of 0.7. A sensitivity analysis was employed in order to select the most appropriate GA parameters, and ensure the solution convergence. The stopping criterion of the NSGA-II algorithm was 0.0001 function tolerance. In this work, three multi-objective models are presented:

(a) Model I (machining performance-based model), only considering the machining outputs (two objectives);
(b) Model II (sustainability performance-based model), only considering the sustainable indicators (two objectives);
(c) Model III (integrated performance-based model), considering both machining outputs and sustainable indicators (three objectives). However, since the power consumption results are directly proportional with the calculated CO_2_ emissions, only three objectives are considered to eliminate any sort of duplication effect.

**Figure 8:**
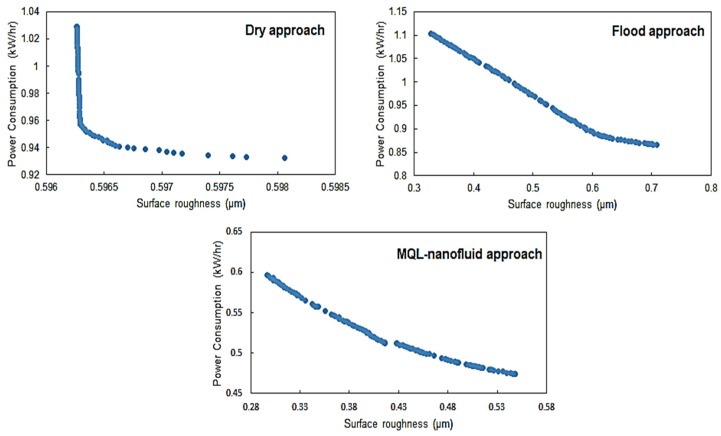
Pareto fronts for Model I of dry, flood and MQL-nanofluid strategies.

**Figure 9:**
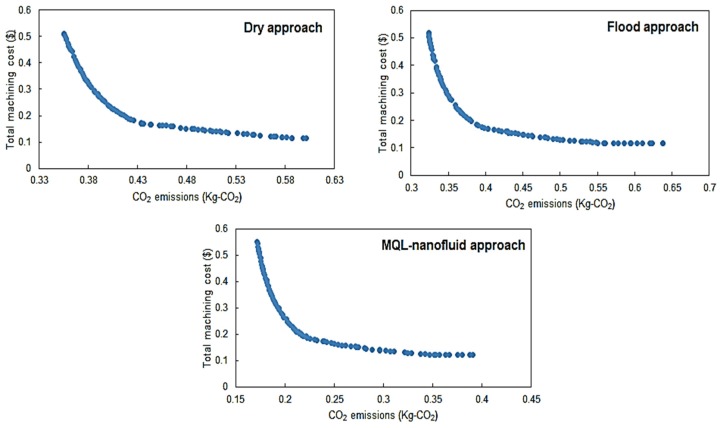
Pareto fronts for Model II for dry, flood and MQL-nanofluid strategies.

**Figure 10:**
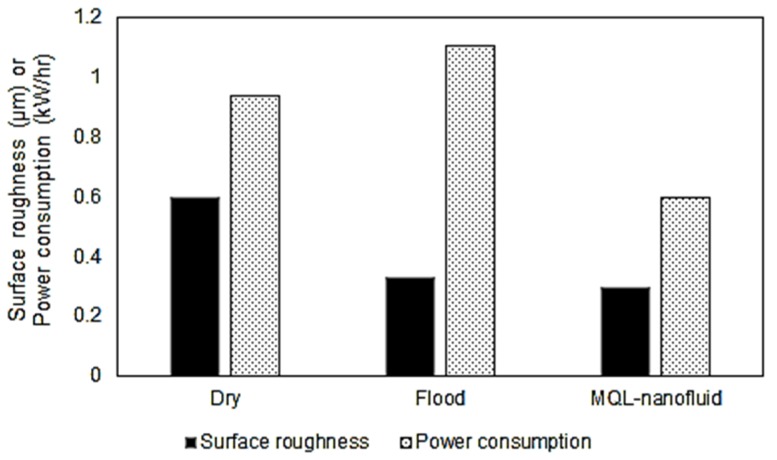
The selected optimal results for Model I (machining performance-based model).

**Figure 11:**
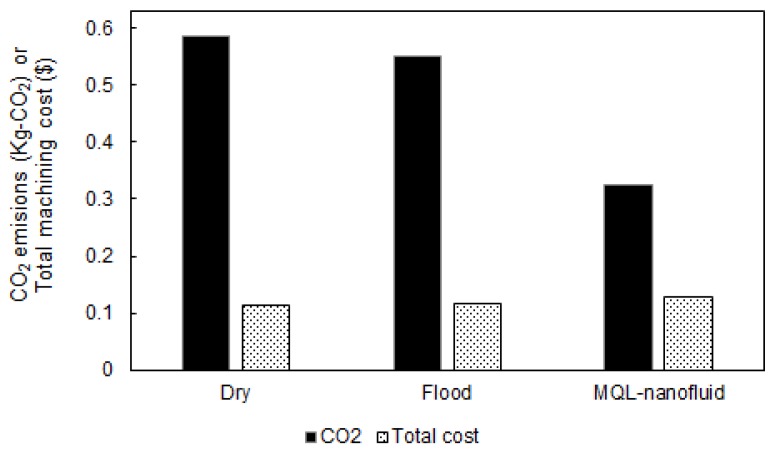
The selected optimal results for Model II (sustainability performance-based model).

The Pareto fronts for both “*Model I*” and “*Model II*” for the three studied strategies are provided in Figure 8 and Figure 9, respectively. For comparing the Pareto fronts, the hyper-volume indicator is applied to measure the dominated part of the volume (space) bounded by the reference point. In addition, the average balanced-effectiveness value is used to select the most effective optimal solution among all solutions provided in each presented Pareto chart. The selected optimal levels achieve a balance between all studied characteristics. It should be stated that Pareto front solutions are all valid to be optimal solutions, and the most appropriate solution is mainly based on the studied responses importance and the highest average balanced-effectiveness value. The selected optimal solutions for Model I and Model II are provided in Figure 10 and Figure 11, respectively.

Regarding the machining performance-based model shown in Figure 10, the optimal conditions of the MQL-nanofluid offered better performance compared to the dry and flood techniques. It should be stated that these values have been selected based on the highest average balanced-effectiveness value. The optimal conditions of the flood offered better performance compared to the dry technique only in terms of induced surface quality; however, the dry offered better performance in terms of the power consumption. The optimal cutting conditions for MQL-nanofluid associated with these optimal responses are: cutting speed of 137 m/min, depth of cut of 0.25 mm and feed rate of 0.06 mm/rev. The optimal conditions for flood technique are cutting speed of 143 m/min, depth of cut of 0.25 mm and feed rate of 0.06 mm/rev; the optimal cutting conditions for the dry technique are cutting speed of 102 m/min, depth of cut of 0.25 mm and feed rate of 0.06 mm/rev. Based on the optimal cutting conditions for the three strategies, It can be concluded that the optimized settings of the MQL-nanofluid based on Model I offered a balanced cutting speed (i.e., 137 m/min) compared to other approaches to achieve the highest possible machining performance.

Regarding the sustainability performance-based model shown in Figure 11, the optimal conditions MQL-nanofluid offered better performance compared to the dry and flood techniques in terms of the CO_2_ emissions, however, dry as well as flood offered a slightly better performance in terms of the total machining costs. In addition, the optimal conditions of the flood offered better performance compared to the dry technique when only considering the CO_2_ emissions, while the dry offered better performance in terms of the total machining costs compared the cutting tests done with flood coolant. The optimal cutting conditions for MQL-nanofluid associated with these optimal responses values are cutting speed of 144 m/min, depth of cut of 0.27 mm and feed rate of 0.18 mm/rev. The optimal conditions for flood technique are cutting speed of 147 m/min, depth of cut of 0.34 mm and feed rate of 0.18 mm/rev; the optimal cutting conditions for the dry are cutting speed of 149 m/min, depth of cut of 0.27 mm and feed rate of 0.18 mm/rev. Similarly, to Model I, the optimized settings of the MQL-nanofluid based on Model II offered a balanced cutting speed (i.e., 144 m/min) compared to other approaches to achieve the highest possible machining performance. In addition, the optimized settings of the MQL-nanofluid offer the same optimal depth of cut (i.e., 0.27 mm) as dry cutting which maintains the productivity efficiency.

**Figure 12:**
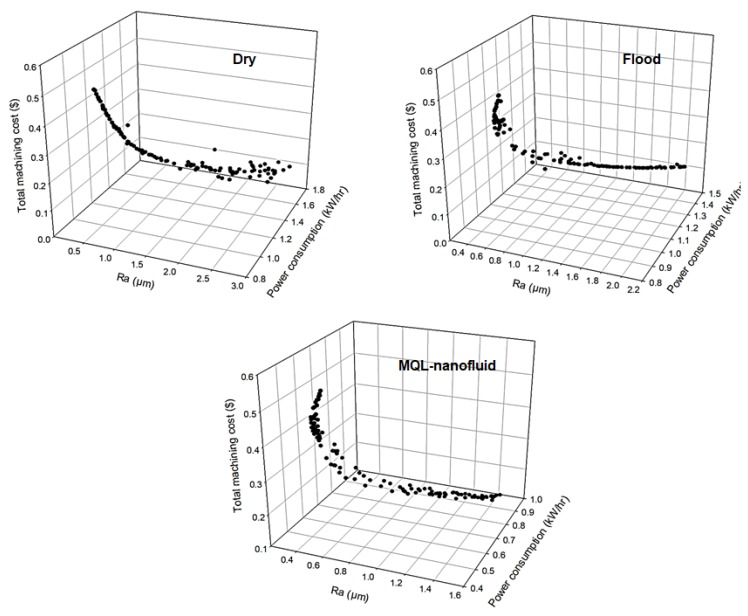
The 3-D Pareto front for Model III.

**Figure 13:**
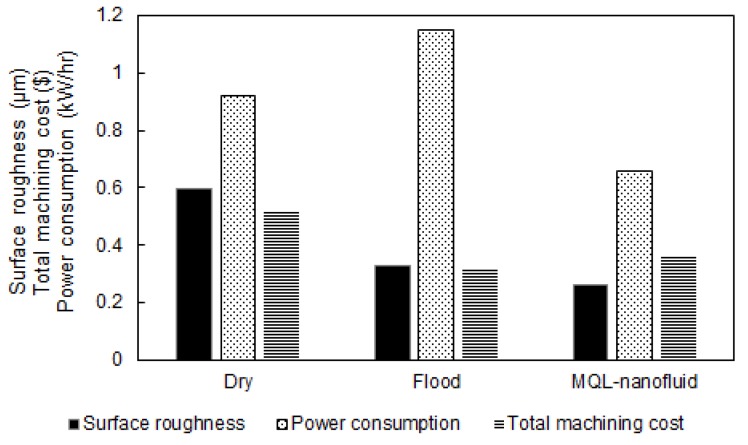
The selected optimal results for Model III (integrated performance-based model).

Regarding Model III (integrated performance-based model), the Pareto front (three objectives) for the three studied strategies is provided in Figure 12. This model includes machining cost, power consumption and surface roughness. The selected optimal results for Model III are provided in Figure 13. The optimal conditions of the MQL-nanofluid offered better performance compared to the dry and flood techniques in terms of surface roughness and power consumption, however, the optimal conditions of the flood techniques offered lowest total machining cost compared to the dry and MQL-nanofluid approaches. It should be also stated that the optimal conditions of the flood approach offered better performance than dry cutting in terms of surface roughness and machining costs; however, better performance is noticed for power consumption under dry cutting compared to the flood technique. The optimal cutting conditions for MQL-nanofluid associated with these optimal responses values are cutting speed of 147 m/min, depth of cut of 0.28 mm and feed rate of 0.06 mm/rev. The optimal conditions for flood technique are cutting speed of 149 m/min, depth of cut of 0.26 mm and feed rate of 0.06 mm/rev; the optimal cutting conditions for the dry are cutting speed of 100 m/min, depth of cut of 0.25 mm and feed rate of 0.06 mm/rev. It can be concluded that the optimized settings of the MQL-nanofluid based on Model III offered an almost similar cutting speed as the flood approach, while it offered higher value of depth of cut (i.e., 0.28 mm) which achieves higher productivity efficiency.

The three studied multi-objective optimization models obtained in this work provide flexibility to the decision maker to select the appropriate cooling/lubrication strategy based on the desired objectives and targets, whether these targets are focused on machining performance, sustainability effectiveness, or both.

## 5. Conclusions and Future Work

This study presents a careful evaluation of turning AISI 1045 steel by applying a robust optimization algorithm based on genetic programming approach correlated to different modern cooling and lubrication strategies, namely dry, flood, and MQL-nanofluids. Three different multi-objective optimization cases have been presented and discussed in this work (i.e., machining performance, sustainability effectiveness, and an integrated model including both). The results gathered in this research are very promising and can be used successfully for industrial application. It should be stated that MQL-nanofluid offered promising results through the three studied cases compared to dry and flood approaches. This is mainly attributed to the cooling capabilities of both flood and MQL-nanofluid techniques to reduce the severity of the high-heat generated in the cutting zone. It should be also stated that MQL-nano-mist does not only offer a promising cooling property, but also it has a pure tribological effect on the performance of the cutting process. The nano-mist serves as rollers in the cutting zone, which affects and improves the friction behavior, and hence a better machining performance was observed when machining under MQL-nanofluid. The results provided in this work offer a clear guideline to select the optimal cutting conditions based on different scenarios. The three studied multi-objective optimization models obtained in this work provide flexibility to the decision maker(s) to select the appropriate cooling/lubrication strategy based on the desired objectives and targets, whether these targets are focused on machining performance, sustainability effectiveness, or both. 

In terms of the future work, more sustainable indicators (i.e., waste management, personal health and safety) can be included to offer a comprehensive sustainable analysis when machining AISI 1045. Moreover, the chip formation behavior and tool wear analysis will be thoroughly discussed in the future to physically understand the machinability performance of AISI 1045 under using these cooling and lubrication approaches. 

## Figures and Tables

**Table 1:** Chemical composition of AISI 1045.

C	Mn	Si	Cu	Cr	Ni	S	Fe
0.45	0.65	0.240	0.20	0.08	0.06	0.003	Balance

**Table 2:** Studied design variables and their levels.

Design Variables	Level 1	Level 2	Level 3
Cutting speed (m/min)	100	125	150
Depth of cut (mm)	0.25	0.50	0.75
Feed rate (mm/rev)	0.06	0.12	0.18
Cooling and lubrication technique	Dry	flood	MQL-nanofluid

**Table 3:** L27 orthogonal array for design of experiments.

Test No.	Cutting Speed, m/min (v)	Depth of Cut, mm (d)	Feed Rate, mm/rev (f)
1	150	0.75	0.18
2	150	0.75	0.12
3	150	0.75	0.06
4	150	0.5	0.18
5	150	0.5	0.12
6	150	0.5	0.06
7	150	0.25	0.18
8	150	0.25	0.12
9	150	0.25	0.06
10	125	0.75	0.18
11	125	0.75	0.12
12	125	0.75	0.06
13	125	0.5	0.18
14	125	0.5	0.12
15	125	0.5	0.06
16	125	0.25	0.18
17	125	0.25	0.12
18	125	0.25	0.06
19	100	0.75	0.18
20	100	0.75	0.12
21	100	0.75	0.06
22	100	0.5	0.18
23	100	0.5	0.12
24	100	0.5	0.06
25	100	0.25	0.18
26	100	0.25	0.12
27	100	0.25	0.06
